# Editorial: Modulating the gut immune microenvironment through nutrients: molecular mechanisms and therapeutic potential in immune-related gastrointestinal disorders

**DOI:** 10.3389/fnut.2026.2011364

**Published:** 2026-10-13

**Authors:** Bowen Li, Francesca Bottacini, Yuhui Yang

**Affiliations:** 1 China-Hungary Belt and Road Joint Laboratory on Food Science, Chongqing Key Laboratory of Specialty Food Co-Built by Sichuan and Chongqing, College of Food Science, Southwest University, Chongqing, China; 2 Department of Biological Sciences, Munster Technological University, Cork, Ireland; 3 College of Food Science and Engineering, Henan University of Technology, Zhengzhou, China

**Keywords:** gastrointestinal disorders, gut immune, inflammatory bowel disease (IBD), microenvironment, nutrients

## 1 Nutrients as upstream tuners of the gut immune microenvironment

Nutrients are increasingly recognized as upstream regulators of the gut immune microenvironment. Dietary components do not simply provide calories or build blocks for host tissues. They determine the substrates available to intestinal epithelial cells, resident microorganisms, and infiltrating immune cells, thereby shaping the molecular conditions under which barrier maintenance, immune tolerance, and inflammatory activation occur. This concept moves beyond the traditional view that diet acts mainly through long term changes in body weight or nutrient deficiency. Instead, dietary carbohydrates, lipids, proteins, micronutrients, and food associated compounds can rapidly modify luminal metabolite pools, oxygen availability, redox state, bile acid composition, and microbial metabolic activity. These local signals directly influence epithelial differentiation, mucus production, tight junction organization, antigen sampling, and the activation state of macrophages, dendritic cells, innate lymphoid cells, and T cells. Recent evidence has emphasized that the metabolic consequences of diet emerge from reciprocal interactions between the host and microbiome, rather than from either component in isolation (1). Thus, nutrients should be viewed as ecological and immunological signals that continually calibrate the intestinal environment.

Dietary fiber illustrates this principle particularly clearly. Fermentable carbohydrates provide substrates for microbial production of short chain fatty acids, including acetate, propionate, and butyrate. These metabolites support colonocyte energy metabolism, reinforce epithelial barrier function, and regulate immune responses through receptor mediated signaling and epigenetic mechanisms. Butyrate oxidation by colonocytes also contributes to the maintenance of the hypoxic intestinal environment that favors obligate anaerobic microorganisms and restricts the expansion of facultative pathogens (2). Conversely, low fiber intake, excessive consumption of refined sugars, saturated fat, red meat, and ultra processed foods may shift the intestinal ecosystem toward reduced SCFA availability, impaired epithelial metabolism, mucus degradation, and inflammatory microbial expansion. This nutritional transition can promote a microenvironment characterized by increased permeability, microbial translocation, and activation of innate immune pathways. Such processes are particularly relevant to inflammatory bowel disease, in which epithelial mitochondrial dysfunction, altered substrate utilization, and immune metabolic may jointly sustain chronic mucosal inflammation (3, 4). More broadly, Western dietary patterns have been linked to chronic disease through convergent effects on metabolism, inflammation, and microbial ecology (5).

The immune consequences of nutrition also extend beyond fiber fermentation. Dietary lipids influence bile acid signaling, membrane composition, lipid mediator generation, and inflammatory cell activation. Amino acids such as tryptophan, glutamine, arginine, and methionine can affect epithelial repair, antioxidant capacity, microbial metabolism, and immune-cell differentiation. In a mouse model of dextran sulfate sodium-induced colitis, 40% dietary methionine restriction ameliorated colonic inflammation and barrier dysfunction, accompanied by gut microbiota remodeling, lower colonic hydrogen sulfide levels, and higher SCFA levels (6). Vitamins and trace elements may regulate redox homeostasis, mitochondrial function, epithelial turnover, and inflammatory signaling. Food processing also changes microbial substrates: thermally processed wheat dough proteins exhibited oxidation, reduced digestibility, and altered human gut microbial composition during *in vitro* fermentation, with baking and air frying producing the most pronounced effects (7). Conversely, adding cyanidin-3-O-glucoside to dough before baking reduced protein oxidation and attenuated the associated microbial shifts during *in vitro* fermentation with human gut microbiota (8). Importantly, these effects depend on timing and context. Meal timing, circadian rhythm, dietary transitions, and prior microbial exposure can all determine how a given nutrient signal is interpreted by the host. Microbiota diurnal oscillations, for example, can program rhythmic host transcriptional responses and connect feeding behavior with immune and metabolic homeostasis (9, 10). Nutritional regulation of the gut immune microenvironment should therefore be understood as a dynamic process involving nutrient quality, dose, combinations, temporal patterns, microbial metabolic capacity, and host susceptibility. This perspective provides the conceptual foundation for the studies assembled in this Topic.

## 2 Thematic advancements: mechanistic elucidation and clinical translation

The contributions in this Topic collectively support a unifying concept that nutrients, dietary patterns, microbial metabolites, and environmental exposures continuously shape the gut immune microenvironment. This microenvironment integrates epithelial metabolism, mucus secretion, tight-junction organization, microbial ecology, and mucosal immune signaling. When these components are coordinated, they sustain barrier integrity and immune tolerance. When disrupted, metabolic stress, dysbiosis, increased permeability, and inflammation can reinforce one another and promote gastrointestinal as well as systemic disease. This framework is particularly evident in the synthesis of metabolic reprogramming in ulcerative colitis, where Western dietary patterns, reduced fiber availability, impaired butyrate utilization, epithelial energy deficiency, immune-cell glycolytic shifts, and microbial dysbiosis form a self-sustaining inflammatory circuit (Li et al.). The accompanying interdisciplinary analysis of intestinal mucosal barrier injury expands this concept beyond gastrointestinal disease, connecting impaired barrier function with nutritional status, environmental exposure, psychological stress, host genetics, and disease processes along the gut-liver, gut-kidney, gut-brain, gut-lung, and gut-skin axes (Wang et al.). Agricultural chemicals represent an additional layer of risk, as pesticides, heavy metals, endocrine-disrupting chemicals, and mycotoxins may converge on common processes that include oxidative stress, microbial disruption, tight-junction injury, and mucosal immune activation (Becherucci et al.).

This overarching framework also extends to non-IBD conditions in which alterations in the gut immune microenvironment may link lifestyle to metabolic or functional disorders. Circadian misalignment induced by irregular feeding, nocturnal light exposure, or disrupted sleep may desynchronize host and microbial rhythms, leading to altered SCFA oscillations, reduced propulsive motility, barrier impairment, and low-grade inflammation in functional constipation (Li et al.). Similarly, obesity-depression comorbidity in college students is associated with exploratory shifts in microbial community composition and predicted metabolic function, supporting the possibility that the microbiota participates in the interplay among adiposity, inflammation, and mental health (Xu et al.). In polyendocrine metabolic ovarian syndrome, the gut microbiota-metabolite-host axis may integrate insulin resistance, androgen excess, inflammation, bile acid signaling, and reproductive dysfunction. Within this context, ketogenic diets and probiotics may have complementary but still unproven effects on metabolic and microbial regulation (Yang et al.). At the population level, stronger adherence to a gut microbiota-supportive dietary pattern is associated with lower colorectal cancer odds and with more favorable inflammatory, permeability, frailty, and psychosocial profiles, emphasizing the preventive potential of microbiota-oriented dietary quality (Song et al.).

Experimental studies in this collection provide mechanistic support for the therapeutic potential of food-derived compounds and nutrient delivery systems. In colitis models, curcumin and quercetin co-delivered in zein–caseinate nanoparticles enhanced SCFA production, improved microbial diversity, restored tight-junction proteins, and suppressed TLR4/NF-κB and JAK2/STAT3 signaling (Li et al.). The combination of quercetin and lutein similarly reduced oxidative stress and inflammatory signaling, improved goblet-cell and barrier function, and remodeled microbial communities in DSS-induced colitis (Xu et al.). Protection against radiation-induced intestinal injury was achieved through blueberry anthocyanins, which reduced oxidative and inflammatory injury, improved epithelial regeneration and Paneth-cell survival, restored tight-junction expression, and reshaped microbial and metabolic profiles. The loss of protection following microbiota depletion and the transfer of benefits by fecal microbiota transplantation strongly support a microbiota-dependent mechanism (Yang et al.). A methionine-enriched diet improved age-related intestinal dysfunction through a microbiota-sulfation axis involving reduced sulfate-reducing bacteria and hydrogen sulfide levels together with restoration of sulfomucin, MUC2, epithelial integrity, and inflammatory balance (Wu et al.). Vitamin K supplementation further demonstrated that micronutrient-based interventions can protect villus architecture, tight-junction proteins, antioxidant defenses, and microbial balance while restraining MAPK and PI3K-AKT signaling during endotoxin-induced intestinal inflammation (Wang et al.).

The remaining contributions emphasize the translational breadth of this field, from precision fiber nutrition to food systems, animal health, and advanced human models. Resistant starch consistently depleted a cross-cohort pathobiont-enriched microbial module that included *Ruminococcus gnavus*, and these resistant starch-depleted taxa were enriched in inflammatory bowel disease cohorts, suggesting a route toward microbiota-informed fiber interventions (Dong et al.). In broilers, dietary *Chinese yam* and *Rehmannia glutinosa* improved antioxidant capacity, immune parameters, slaughter performance, and cecal microbiota composition (Dong et al.). In crucian carp, selected *Latilactobacillus sakei* strains protected against *Aeromonas hydrophila*-induced injury by reducing inflammatory activation, supporting mucosal immunity, and reconfiguring microbial communities (Zhao et al.). Tea leaves affected by blister blight, traditionally viewed as an agricultural loss, were shown to contain a bioactive-rich aqueous extract capable of reducing copper-induced oxidative stress, inflammation, and apoptosis in zebrafish embryos (Liu et al.). Finally, a dual-porosity electrospun platform supporting direct interactions among primary intestinal epithelial cells, dendritic cells, and T cells provides a human-relevant experimental system for testing how nutrients, microbial metabolites, allergens, and pathogens influence the mucosal immune interface (Walker et al.). Together, the articles move the field toward a precision framework in which nutritional strategies are evaluated not only by their composition, but also by their ability to restore microbial function, epithelial resilience, and immune homeostasis.

## 3 Toward causal mechanisms and precision nutrition

Building on the evidence summarized above, the next challenge is to move from association to causation in nutrient-microbiota-immune research. Altered microbial taxa, metabolites, epithelial markers, and inflammatory pathways are increasingly reported across immune-related gastrointestinal disorders, yet it often remains unclear whether these changes initiate disease or arise secondarily from inflammation, medication use, and altered dietary intake. Future studies should therefore integrate longitudinal cohorts with controlled nutritional interventions and repeated sampling to define the temporal sequence linking nutrient exposure, microbial remodeling, metabolite production, barrier adaptation, and immune activation. Combining metagenomics, metabolomics, host transcriptomics, and immune profiling with mechanistic validation in organoids, epithelial-immune co-cultures, gnotobiotic models, and fecal microbiota transplantation systems will be essential to identify the microbial functions and host pathways that are truly causal.

Establishing such causal and mechanistic frameworks is also the prerequisite for precision nutrition. Individual responses to fibers, probiotics, amino acids, polyphenols, vitamins, or ketogenic dietary approaches are likely to depend on baseline microbiota composition, microbial functional capacity, SCFA and bile acid profiles, intestinal permeability, inflammatory status, metabolic phenotype, medication exposure, and habitual diet. Future clinical trials should therefore incorporate standardized intervention protocols, robust adherence assessment, safety monitoring, and mechanistic endpoints alongside conventional clinical outcomes. By linking these multidimensional profiles with treatment response, nutritional interventions may be matched more rationally to disease phenotype and biological context. Ultimately, this approach could support the integration of targeted nutrition with established therapies to restore epithelial resilience, microbial function, and immune tolerance.
